# Impact of chemotherapeutic agents on liver microenvironment: oxaliplatin create a pro-metastatic landscape

**DOI:** 10.1186/s13046-023-02804-z

**Published:** 2023-09-11

**Authors:** Yuanyuan Ma, Chang Guo, Xijun Wang, Xundong Wei, Jie Ma

**Affiliations:** 1 Center of Biotherapy, Beijing Hospital, National Center of Gerontology; Institute of Geriatric Medicine, Chinese Academy of Medical Sciences, Graduate School of Peking Union Medical College, Beijing, 100730 People’s Republic of China; 2 Savaid Medical School, University of Chinese Academy of Sciences, Beijing, 100049 People’s Republic of China

**Keywords:** Chemotherapeutic agents, Colorectal cancer, Liver metastasis, Pre-metastasis microenvironment, T cell crosstalk

## Abstract

**Background:**

Chemotherapeutic agents are used to control tumor proliferation. However, their influence in the pre-metastatic niche of target organs has not been well studied. Oxaliplatin (OXA) is a drug applied in standard treatments of colorectal cancer (CRC), while the direct effect of which on the pre-metastatic microenvironment of the liver remains unclear.

**Methods:**

Models of liver metastases were established with luciferase expressing CT26 cells in BALB/c and BALB/c-nude mice. Single-cell RNA Sequencing was performed to examine the immune microenvironment in the liver elicited by OXA. Immunofluorescence and flowcytometry were utilized to confirm the changes in the number of immune cells. LDH, CellTrace CFSE Cell Proliferation and apoptosis assays were conducted to explore the impact of OXA on T cells ex vivo. The correlation between chemotherapy-related lymphopenia and metastases was assessed by meta-analysis.

**Results:**

Herein we discovered that administration of OXA prior to the occurrence of liver metastasis actually accelerated tumor development and colonization in the liver. Single-cell RNA sequencing revealed that the landscape of the liver immune microenvironment had been changed to immunosuppressive phenotype. Macrophages after the treatment of OXA exhibited a high ability to inhibit the activation of T cells. Further investigation revealed a significant decrease in the number of T cells in the liver, particularly CD8^+^ T cells with reduced capacity of proliferation, activation, and killing. When mice were treated with T cell supplementation, the OXA-induced metastasis was notably abolished, indicating that the OXA-primed liver microenvironment could be reversed by the infusion of T cells. Consistent with our findings in mice, a meta-analysis was performed to verify that chemotherapy-related lymphopenia was associated with an inferior prognosis related with high incidence of metastasis, suggesting the pivotal role of chemotherapy in pre-metastatic niche formation. Furthermore, a notable reduction in the count of both macrophages and T cells was observed in the liver of colorectal cancer (CRC) patient undergoing OXA-based chemotherapy.

**Conclusions:**

Our findings proposed that immunosuppressive microenvironment in liver induced by OXA enhanced liver metastasis of colorectal cancer, which highlighted a new consideration to balance the pro metastases and anti-cancer possibility of OXA treatment.

**Supplementary Information:**

The online version contains supplementary material available at 10.1186/s13046-023-02804-z.

## Background

Colorectal cancer (CRC) is the third most common cancer worldwide which ranks second in terms of mortality [1]. The presence of liver metastasis, even at early stages or at diagnosis, is a significant factor contributing to the poor prognosis of CRC patients. Chemotherapy is considered the cornerstone of cancer treatment, as it can reduce unresectable metastases and improve progression-free survival [2]. Combination therapies such as FOLFOX (5-FU/leucovorin and oxaliplatin) and FOLFIRI (5-FU/leucovorin and irinotecan) have proven to be effective cytotoxic regimens for treating metastatic CRC [3]. FOLFOX is widely accepted as the standard adjuvant chemotherapy as well as neoadjuvant chemotherapy [4]. However, besides the common adverse reactions of chemotherapy such as fatigue, digestive system symptoms, and peripheral neuropathy [5], the increased use of chemotherapy for colorectal liver metastases (CRLM) has raised concerns about potential hepatotoxicity leading to sinusoidal injury [6].

Oxaliplatin (OXA), a third-generation platinum chemotherapeutic agent, is widely used for neoadjuvant and adjuvant treatment of CRC. OXA-based chemotherapy, such as FOLFOX, has been recommended as first-line chemotherapeutic drug because of improved resection possibility and outcomes. However, OXA-induced liver injury, sinusoidal obstruction syndrome (SOS), remains a major limitation of OXA-based chemotherapy in patients with CRLM [7]. OXA-induced SOS is a distinct drug-specific side effect characterized by damage, necrosis, and detachment of endothelial, and microthrombi in hepatic sinusoids. SOS results in the dilation, rupture, and bleeding of hepatic sinuses, as well as inflammatory cell infiltration and varying degrees of hepatocyte necrosis. In later stage, the disease can progress to liver fibrosis and cirrhosis [8]. Although SOS has been reported to promote CRLM development in animal models [9], the underlying mechanism is not clear. Therefore, whether this side effects of OXA will increase the risk of liver metastasis worth well study.

In addition to SOS, there have been reports about modification of microenvironment of remote organs by chemotherapy. For instance, Keklikoglou et al. demonstrated that neoadjuvant chemotherapy related tumor-derived extracellular vehicles (EVs) promoted endothelial cell activation, CCL2 production, and Ly6C^+^CCR2^+^ monocyte expansion in the pulmonary pre-metastatic niche, which eventually facilitated the establishment of lung metastasis [10]. In another study, Daenen et al. discovered that cisplatin and paclitaxel increased VEGFR1 expression in lung endothelial cells, facilitating the retention of circulating tumor cells in the metastatic site [11]. These findings hint that systemic administration of OXA might also induce the formation of local pro-metastatic niche besides SOS.

In this study, we aimed to explore the effects of OXA-induced microenvironment changes in the liver. Our findings revealed that pretreatment with OXA enhanced liver metastasis formation which is attributed to a dramatic decrease in total number of macrophages and T cells but increase in composition of immunosuppressive macrophages. Moreover, supplement with T cell reconstructed OXA-primed liver microenvironment leading to improved metastatic situation in mice. Taken together, our study indicated that OXA-associated chemotherapies contribute to the construction of pro-metastatic microenvironment in the liver, which could be repaired by adoptive cell therapy.

## Methods

### Cell lines and mice model

Murine colon carcinoma cell line CT26luc cells were purchased from Biohelix Biotech Co. Ltd (Guangzhou, China). Cells were grown at 37 °C in a humidified atmosphere containing 5% CO_2_ and incubated in RPMI-1640 medium (Hyclone) supplemented with 10% fetal bovine serum (FBS, Gibco).

BALB/c and BALB/c-nude (female, six to eight weeks of age) were purchased from Beijing Huafukang Bioscience Co. Ltd, maintained in microisolator cages under specific pathogen-free (SPF) conditions at the Cancer Hospital Chinese Academy of Medical Sciences (Beijing, China). All animal experiments were performed in accordance with the guidelines of the Laboratory Animal Ethics Committee of Cancer Hospital Chinese Academy of Medical Sciences (Permit Number NCC2021A296).

CT26 hepatic metastases model were constructed by spleen injection of 1.0 × 10^6^ CT26luc tumor cells. To assess the effect of OXA pre-treatment on cancer metastasis, mice were given 15 mg/kg oxaliplatin or PBS intravenously three days before intrasplenic inoculation of CT26luc cells. At the end point, twenty minutes before photon recording, the mice were intraperitoneally injected with 200 μl of D-luciferin sodium salt (15 mg/ml), and then euthanized. The livers were dissected and imaged by a cooled charged-coupled device (CCD) camera to detect the fluorescence intensity.

For T cells infusion treatment, we first prepared T-cell suspensions by isolating single-cell suspensions from splenocytes using 70-µm cell strainers. Cells were activated on plates coated with 2 mg/ml of anti-mouse CD3e and 1 mg/ml of CD28 in T cells culture medium (RPMI 1640, 10% FBS, and 100 U/ml of mouse IL-2) for 48 h at 37 °C, and grew further in T cells culture medium for 5 days. The prepared T-cell suspensions were injected into the mice at a concentration of 10^7^ per mouse at the time points shown in the treatment model.

### Patients and specimen collection

Resected surgical liver tissues of CRC patients (n = 24) were collected from Cancer Hospital, Chinese Academy of Medical Sciences. The use of pathological specimens and the review of all patient clinical records were approved by the Ethics Committee of National Cancer Center/ Cancer Hospital, Chinese Academy of Medical Sciences and Peking Union Medical College (project approval number 22/130 - 3331).

### Measurement of Serum Alanine Aminotransferase (ALT) and Aspartate Transaminase (AST)

The concentrations of serum ALT and AST were assessed using the biochemistry kit from Solarbio Life Science (Beijing, China), following the manufacturer's instructions.

### Single-cell RNA sequencing (scRNA-seq) and data processing

The liver collected from mice treated with OXA three days prior was dissociated into single-cell suspensions using the Liver Dissociation Kit (mouse) from Miltenyi Biotec (Germany). Single-cell library preparation was performed using Chromium Single Cell 3' reagent kits v2, and sequencing was carried out on the Illumina Hiseq X-Ten platform provided by Majorbio Corporation (Shanghai, China). The raw gene expression matrices were analyzed using R software (version 4.2.0) with the Seurat package (version 4.1.1) [12] and merged for further analysis. Low-quality cells were excluded if they met any of the following criteria: cells with fewer than 200 or more than 3000 detected genes, or cells with a high fraction of mitochondrial genes (> 20%).

Next, the gene expression data were normalized, and the top 2000 most variable genes were calculated using the 'FindVariableFeatures' function. The data were then scaled using the 'ScaleData' function, and the 'RunPCA' function was applied with default parameters to reduce dimensionality. The 'FindNeighbors' and 'FindClusters' functions were used to identify cell clusters, with a resolution parameter set to 0.8. Subsequently, the t-distributed stochastic neighbor embedding (t-SNE) was employed to visualize the clustering results in a nonlinear dimensional reduction manner.

### Cell type annotation and cluster marker identification

After projecting all cells into two-dimensional spaces using t-SNE, cells were clustered based on the expression of cell type-associated genes reported in the literature. Signature genes of each cell cluster were identified using the Seurat 'FindAllMarkers' function. To further decipher transcriptomic changes with OXA treatment, the T cells were further classified into different subtypes using the same procedures.

### Differential gene expression and functional enrichment

The 'FindAllMarkers' function was utilized to identify differentially expressed genes (DEGs) with the default parameters. DEGs were filtered based on log2(fold change) > 0.25 and false discovery rate (FDR) < 0.05. Functional enrichment analysis of DEGs was performed using the Metascape webtool (https://metascape.org).

### Defining cell feature scores and cell cycle state

For cell feature scores calculation, the Seurat 'AddModuleScore' function was employed, and positive regulation of immune effector process (GO: 0002699), positive regulation of T cell activation (GO: 0050870) and negative regulation of T cell activation (GO: 0050868) were used to define immune effector and activation scores, respectively. For cell cycle assessment, the Seurat 'CellCycleScoring' function was utilized to predict the stage of each cell in the G1, S, or G2M phase, reflecting the cell proliferation ability.

### Flow cytometry

Mice were euthanized, and the liver and lung were harvested and minced into small pieces at 4 °C in Hank's Balanced Salt Solution (HBSS) containing collagenase IV (1 mg/mL, Worthington) and DNase I (150 U/mL, Roche) [13]. The tissues were then incubated at 37 °C for 30 min with intermittent agitation and filtered through 70 μm nylon strainers (Corning). The prepared cell suspensions were incubated with antibodies against murine cell surface markers, including CD16/CD32, CD45 (30-F11), CD3e (17A2), CD4 (GK1.5), CD8a (53–6.7), B220 (RA3-6B2), Ly6C (HK1.4), Ly6G (1A8), and F4/80 (BM8), purchased from BioLegend, and CD11b (M1/70) purchased from Invitrogen. For intracellular staining, the BD Cytofix/Cytoperm Fixation/Permeabilization Kit (BD) was used after surface staining, followed by the staining against: IFNγ (XMG1.2), TNFα (MP6-XT22) and CD206 (C068C2). The data were analyzed using Flowjo v.10 software (USA) as previously described [14].

### Immunofluorescence staining

Liver tissue sections were deparaffinized through dimethylbenzene and rehydrated in alcohol gradients. Antigen retrieval was performed using EDTA pH 9.0 buffer at 100 °C for 10 min. Primary antibodies used were as follows: anti-mouse/human CD45 (1:2000, ab208022, Abcam), anti-mouse CD3 (1:500, 17,617–1-AP, Proteintech), anti-human CD3 (1:200, 85061 T, CST), anti-mouse CD19 (1:1000, ab245235, Abcam), anti-mouse F4/80 (1:1000, 28,463–1-AP, Proteintech) and anti-human CD68 (1:5000, ab213363, Abcam). The sections were incubated with primary antibodies overnight at 4 °C. Subsequently, the primary antibodies were detected by incubating the sections with CoraLite488-conjugated secondary antibody (1:500; Proteintech) or CoraLite594-conjugated secondary antibody (1:500; Proteintech) for 1 h at room temperature. The nuclei were stained with 4',6-diamidino-2-phenylindole (DAPI), and images were captured using a Zeiss fluorescence microscope.

### Cell proliferation analysis and killing assays

Cell proliferation of T cells was quantified using the CellTrace CFSE Cell Proliferation Kit (Invitrogen) according to the manufacturer's instructions. T cells were labeled with CFSE reagent before activation and incubated with increasing concentrations of OXA (0–10 µM) in T cell culture medium for 3 days after activation. The fluorescence intensity excited by 488 nm was detected using the BD FACSVerse Cytometer. For killing assays, murine T cells and human T cells (3 days after activation) were plated in round-bottomed 96-well plates with 10^4^ CT26 and HT29 cells per well, respectively, at the CTL-to-target ratios shown. For OXA treatment, T cells were subjected to 5 µM OXA 3 days prior to co-culture. The co-cultures were incubated at 37 °C for 10 h, and the percentage of target cell lysis was determined using the Cytotoxicity LDH Assay Kit-WST (Dojindo) following the manufacturer's instructions.

### Apoptosis experiment

T-cell suspensions were prepared by isolating single-cell suspensions from splenocytes and activated in T cells culture medium for 2 days. Then, T cells were treated with increasing concentrations of OXA (0–20 µM) in T cell culture medium for 3 days after activation. After that, the cells were harvested and managed using Annexin V, FITC Apoptosis Detection Kit (Dojindo) following the manufacturer's protocol.

### Co-culture and stimulation of T cells

For the co-culture of macrophages and T cells, mouse livers were collected on day 3 after PBS or OXA administration. Macrophages were isolated using Percoll density gradient centrifugation. T cells were obtained by isolating single-cell suspensions from splenocytes, followed by in vitro activation with 2 μg/mL plate-bound anti-CD3 (clone 145–2C11, Biolegend), along with macrophages. Subsequently, 5 × 10^4^ T cells were incubated with 2 × 10^5^ macrophages in a 24-well plate for 72 h for further analyses.

### Meta-analysis data collection and statistical analyses

Meta-analysis was conducted following the Preferred Reporting Items for Systematic Reviews and Meta-Analyses (PRISMA) 2020 guidelines [15]. Studies were searched from PubMed, Embase, Web of Science, and the Cochrane Library, with the search updated to April 5, 2023. Included studies met the following criteria: (1) a cohort study including cancer patients who underwent chemotherapy as part of their treatment; (2) recording lymphopenia information after chemotherapy; (3) providing lymphopenia-related Hazard Ratio (HR) and 95% Confidence Interval (95%CI) for outcomes such as overall survival (OS), progression-free survival (PFS), and metastasis-free survival (DMFS). Studies with fewer than 10 patients, non-English language, or published before 2010 were excluded.

The quality of the final selected studies was evaluated using the Newcastle–Ottawa Quality Assessment Form for Cohort Studies (NOS) [16]. Information extracted from each study included the first author/publication year, country, tumor type, number of patients, age, study design type, treatment information, cut-off value of lymphocyte counts for comparison, and definition of lymphopenia or data collection time. The review process, data extraction, and quality assessment were performed by two independent investigators, with disagreements resolved by three reviewers.

Meta-analyses were performed using R 4.0.2 software (http://www.R-project.org; 'meta' package). Pooled HR and 95% CI of OS, PFS, and DMFS were calculated using the generic inverse variance method with the "metagen" function. Both univariate and multivariate results were included. Statistical heterogeneity was evaluated using the I^2^ test, and publication bias was assessed using Begg's test with the "metabias" function. Sensitivity analyses were performed to detect potential sources of heterogeneity using the "metainf" function. Forest plots were used to visually display results of individual studies and synthetic results. A *p*-value of less than 0.05 was considered statistically significant.

### Statistical analysis

Statistical analysis was conducted using SPSS 25.0 and GraphPad Prism 9. Results were presented as the mean ± SD. Significant differences were determined using one-way ANOVA and two-tailed unpaired Student’s t test: * *P* < 0.05, ** *P* < 0.01, *** *P* < 0.001.

## Results

### Oxaliplatin pre-administration increases liver susceptibility to metastatic colonization of colorectal cancer

**Fig. 1:**
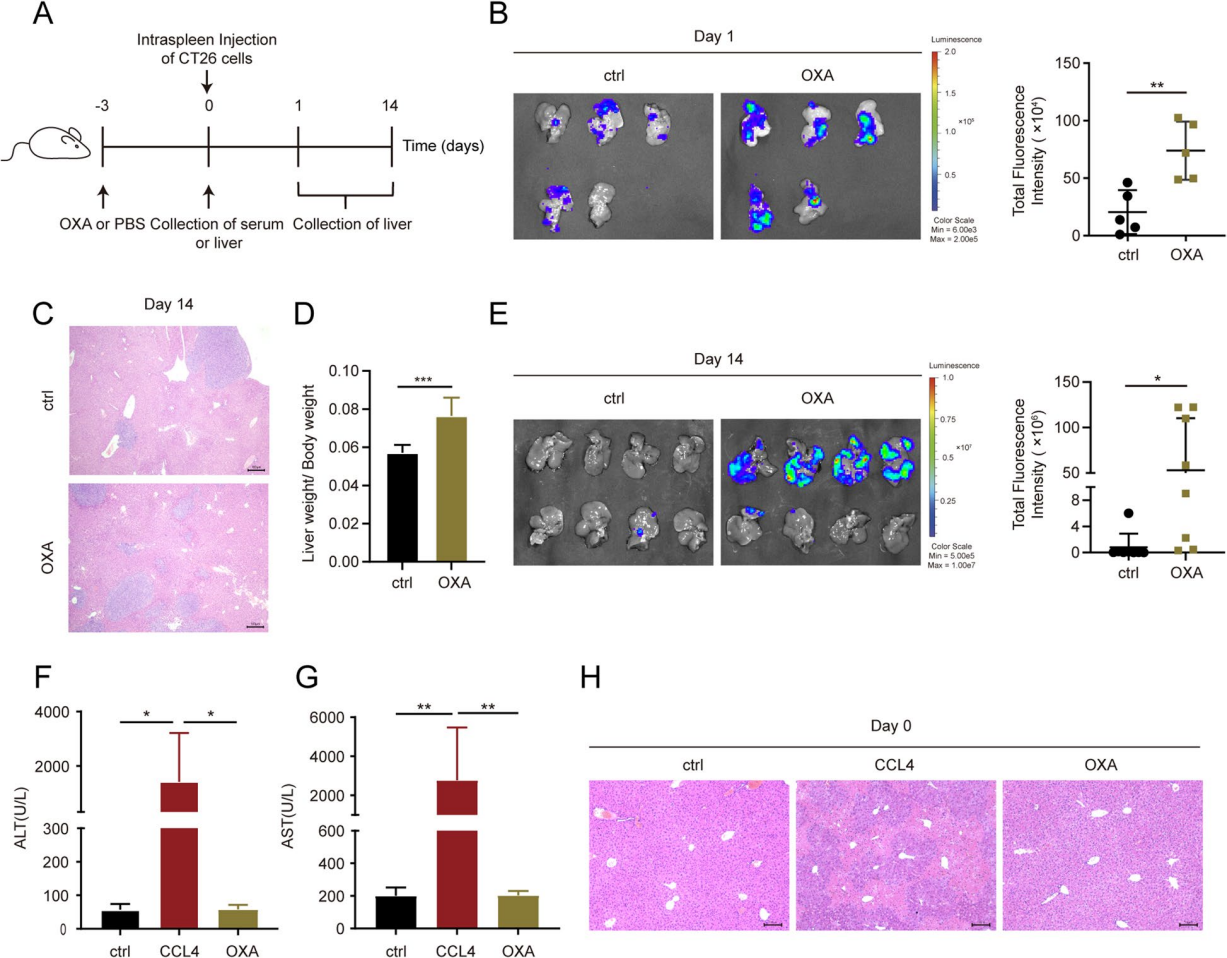
Oxaliplatin pre-administration increases liver susceptibility to metastatic colonization of colorectal cancer. (**A**) Schematic diagram of OXA administration in CT26luc liver metastasis model. Female BALB/c mice were randomly divided into two groups. OXA was intravenously administered to mice three days before intrasplenic inoculation of CT26luc cells (OXA); control mice were treated with PBS (ctrl). Sacrificed on day 1 or day 14 after CT26luc cells inoculation. (**B**, **E**) Luminescence intensities of CT26luc liver metastasis from mice treated with PBS or OXA on day 1 (**B**) or day 14 (**E**) after CT26luc cells inoculation. Left: Representative liver images. Right: Dot plot showing the metastatic burden in the liver (mean ± SD, *n* = 5 to 8 mice). (**C**) Representative HE images of mouse liver tissues displayed the number of metastases on day 14 (Scale bars, 100 µm). (**D**) Metastasis indexes showed that OXA treatment significantly increased liver metastasis in BALB/c mice (mean ± SD, *n* = 5 to 8 mice). (**F**, **G**) Analysis of mouse blood ALT (**F**) and AST (**G**) levels 3 days after OXA administration (day 0), with CCL4 as positive control (mean ± SD, *n* = 5 to 7 mice). (**H**) Representative HE images of liver tissues displayed no obvious changes in the structure between OXA and control group on day 0. Significant differences were assessed using one-way ANOVA and two-tailed unpaired Student’s t test. * *P* < 0.05, ** *P* < 0.01, *** *P* < 0.001

To investigate the impact of pre-administration of oxaliplatin (OXA) on tumor liver metastasis, we established a CT26 hepatic metastasis model by injecting cells into the spleen (Fig. 1A). As depicted in Fig. 1B, intravenous injection of OXA prior to the inoculation of luciferase-transfected CT26 (CT26luc) cells resulted in a significant increase in hepatic retention after 24 h (*P* < 0.01). Consistently, mice pre-injected with OXA exhibited a notable increase in liver metastasis at day 14 following cancer cell inoculation (Fig. 1C-E). Besides OXA, 5-Fluorouracil (5-Fu) is also widely used for the treatment of colorectal cancer [17]. To comprehensively investigate the presence of analogous effects with 5-Fu, mice were administered with 5-Fu intravenously prior to tumor inoculation (Supplementary Fig. S1A). Nonetheless, no substantial changes were observed when mice pretreated with 5-Fu (Supplementary Fig. S1B-C). Previous studies have suggested that OXA-based chemotherapy may induce liver injury. To determine if liver injury contributes to the pro-metastatic effect of OXA, we assessed the activity of Alanine Aminotransferase (ALT) and Aspartate Transaminase (AST) in serum. However, no significant differences in enzyme activity were observed between the OXA-treated and PBS-treated groups (Fig. 1F-G), indicating that a single injection of OXA did not result in significant liver injury in our hepatic metastasis model. Furthermore, there were no significant alterations in the hepatic sinusoidal structure in mice receiving OXA injection (Fig. 1H). Collectively, these findings indicate that the pre-administration of OXA enhances experimental colon cancer metastasis to the liver, and this effect is independent of liver injury.

### scRNA-seq profling of mouse livers ecosystem upon OXA treatment

**Fig. 2:**
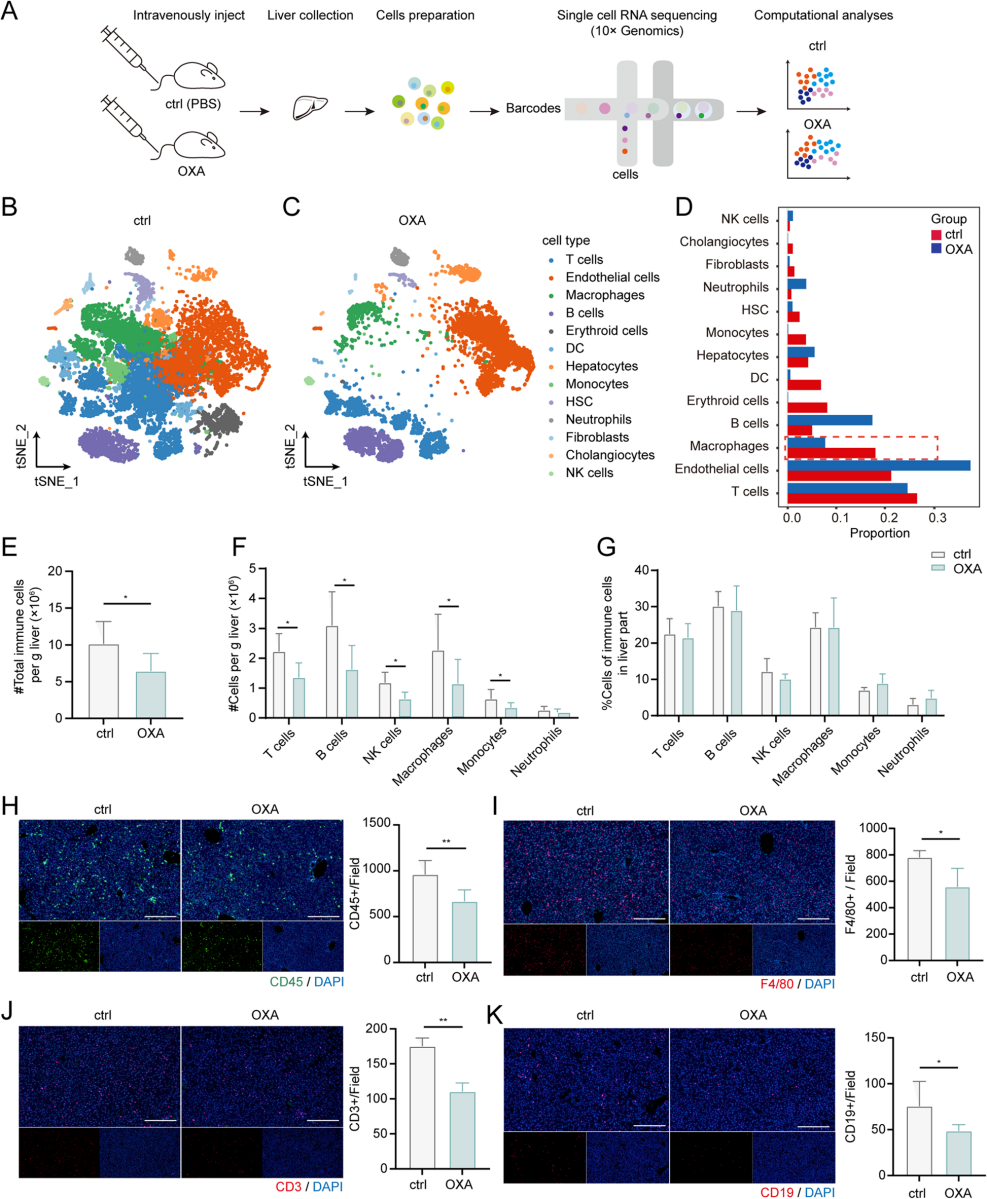
Changes in cell composition of the mice liver after OXA treatment. (**A**) Schematic diagram of the experimental design, scRNA-seq and data analysis. (**B**, **C**) tSNE projections of liver cells in mice treated with PBS (**B**) and OXA (**C**) based on single-cell transcriptomes. Each dot represents a single cell; colors indicate cell clusters with labels. (**D**) Bar plot showing the changes in the percentage of each cell upon OXA treatment. (**E)** Flow cytometry qualification of CD45 + immune cells in livers from control and OXA group (*n* = 6 to 8 mice). (**F**, **G**) Flow cytometry qualification of the number (**F**) and frequency (**G**) of different immune cell populations in livers from mice after 3-day PBS or OXA treatment (*n* = 6 to 8 mice). (**H**–**K**) Representative immunofluorescence analyses of CD45 (left, **H**), F4/80 (left, **I**), CD3 (left, **J**) and CD19 (left, **K**) in livers from mice after 3-day PBS or OXA treatment and quantification analyses of positive cells displayed in the right (*n* = 6 to 8 mice; at least three random fields were selected, mean ± SD, Scale bars, 100 µm). Significant differences were assessed using one-way ANOVA and two-tailed unpaired Student’s t test. *p < 0.05, ** *P* < 0.01

To gain further insights into the mechanism by which OXA pre-administration promotes liver metastasis, we collected liver samples from mice treated with PBS or OXA for 3 days and performed single-cell transcriptomic analysis of liver cells (Fig. 2A). After quality control and filtering, we obtained a total of 28,075 cells (20,162 from the PBS-treated group, ctrl group; 7,913 from the OXA-treated group, OXA group) and classified them into 13 cell lineages (Fig. 2B-C) based on the expression of cell type-associated genes (Supplementary Fig. S2A-B). These lineages included T cells (*Cd3d*, *Cd3g*), endothelial cells (*Ushbp1*, *Dpp4*), macrophages (*Cd68*, *Clec4f*), B cells (*Cd79a*, *Cd22*), erythroid cells (*Hbb-bt*, *Hbb-a1*), dendritic cells (DCs) (*Siglech*, *Irf8*), hepatocytes (*Apoa1*, *Ass1*), monocytes (*S100a8*, *S100a9*), HSC (*Lrat*, *Reln*), neutrophils (*Cxcr2*, *Lcn2*), fibroblasts (*Col1a1*, *Col3a1*), cholangiocytes (*Epcam*, *Spp1*), and natural killer (NK) cells (*Nkg7*, *Itga2*).

Previous studies have implicated the injury of hepatic sinusoidal endothelial cells as a key mechanism underlying OXA-induced sinusoidal obstruction syndrome (SOS), which is closely associated with patient prognosis. Therefore, we examined the effect of OXA on endothelial cells and found no detectable impact on genes associated with endothelial dysfunction and vascular injury, including *Fabp4*, *Pcdh17*, *Esm1*, and *Cd34* (Supplementary Fig. S2C).

Importantly, various immune cells exhibited a lower proportion in the OXA-treated group, including T cells, DCs, and monocytes, particularly macrophages (Fig. 2D). This observation was further confirmed through flow cytometry analysis, which showed significant reductions in the numbers of total immune cells, T cells, macrophages, and monocytes in the OXA-primed liver (Fig. 2E, F). Immunofluorescence assessment also supported these findings (Fig. 2H-J). However, there were no significant changes in the fraction of various immune cells within CD45 + cells following OXA treatment in mice (Fig. 2G), suggesting that the effect of OXA on different immune cells is consistent. Flow cytometry analysis additionally confirmed decreases in the numbers of B cells and NK cells upon OXA treatment (Fig. 2F), which were not evident in the single-cell RNA-seq data. Furthermore, we collected liver samples from mice treated with either PBS or 5-Fu three days prior to examine alterations in the cellular composition of both macrophages and T cells. In contrast to the effects of OXA, the administration of 5-Fu did not induce a noteworthy reduction in immune cells (Supplementary Fig. S1D-F), thereby offering a partial explanation for the absence of liver metastasis in mice pretreated with 5-Fu.

Furthermore, in addition to changes in the numbers of liver cells, we investigated the effects of OXA on the transcriptomic profiles of all liver cells between mice with and without OXA administration. The analysis of differentially expressed genes (DEGs) revealed downregulation of genes associated with the regulation of lymphocyte activation and immune effector processes (Supplementary Fig. S2D, E). This suggests that transcriptomic changes in T cells may contribute to the acceleration of liver metastasis following OXA treatment. Collectively, these findings indicate that OXA treatment reduces the numbers of multiple immune cells, especially macrophages and T cells, in the liver and alters transcriptomic features related to lymphocyte activation and immune effector processes, which may account for the promotion of liver metastasis.

### Macrophages exhibited a more immunosuppressive phenotype in OXA-primed livers

**Fig. 3:**
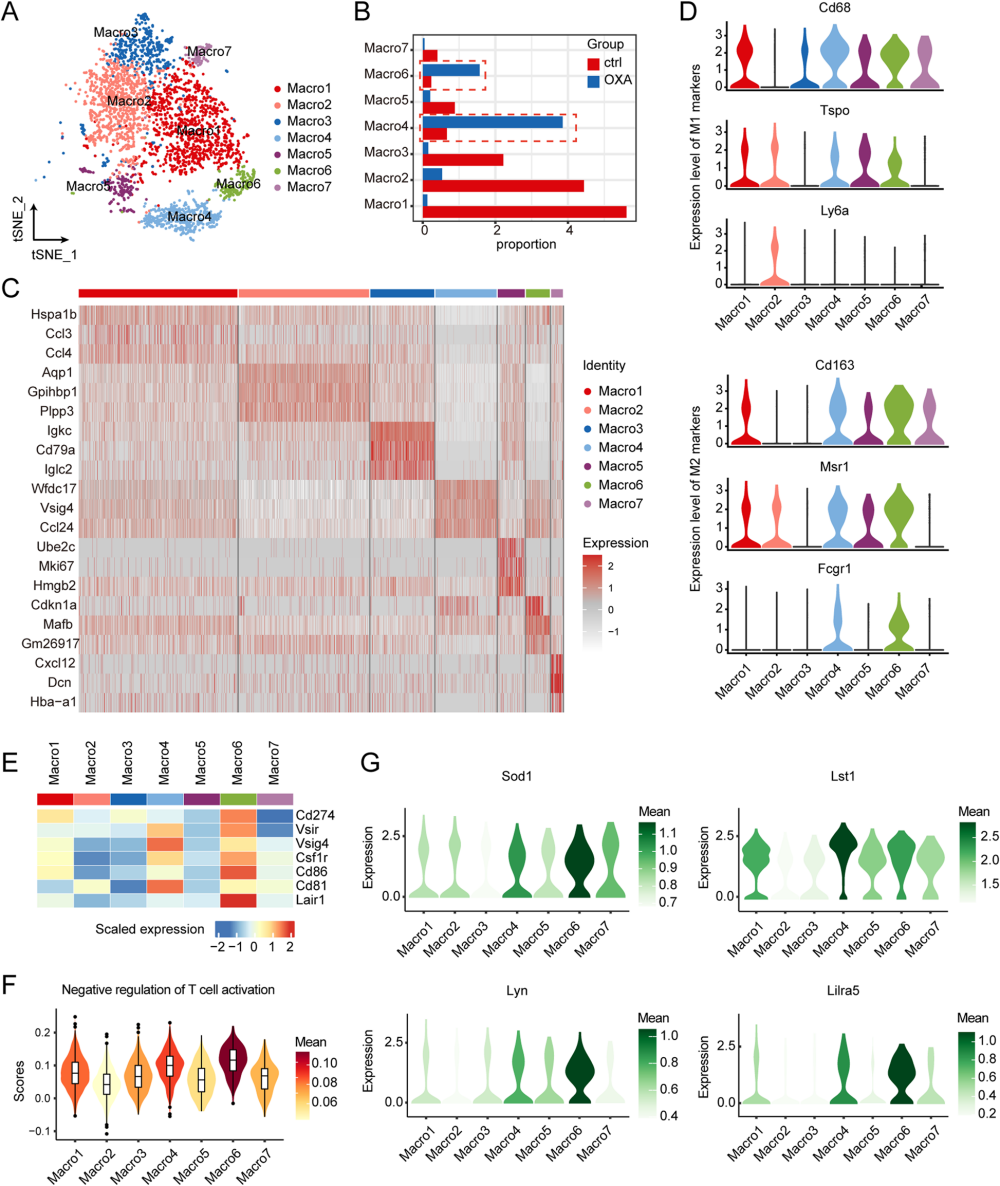
Characterization of macrophages in livers from mice treated with or without OXA. (**A**) t-SNE plot showing the subtypes of macrophages derived from control and OXA samples. Each cluster is color-coded according to the cell type. (**B**) Bar plot showing the changes in the percentage of subpopulations of macrophages upon OXA treatment. (**C**) Heatmap representing scaled expression of top 3 DEGs of the subclusters in macrophages. (**D**) Violin plots showing the expression levels of M1 and M2 marker genes in macrophage subtypes. (**E**) Heatmap showing the scaled expression of genes associated with immune checkpoint and evasion in each cell type. (**F**) Violin plots showing the functional scores (negative regulation of T-cell activation) of macrophages subtypes. (**G**) Violin plots representing the expression of immunoregulatory genes in macrophages subtypes

Innate immune cells, particularly macrophages, play a critical role as the initial defense against the rapid dissemination of cancer cells in the intravascular space. Therefore, we first investigated the impact of OXA on the transcriptomic profile of macrophages, which significantly decreased in mice receiving OXA administration. Subsequently, we analyzed the transcriptomes of 3,426 macrophages, with 2,913 from the control group (ctrl) and 513 from the OXA group. These macrophages exhibited remarkable heterogeneity and were classified into seven subpopulations (Fig. 3A-C). The most noteworthy observation was the remarkable increase in the proportion of Marco4 and Marco6 subpopulations in the OXA group, in contrast to the higher abundance of Marco1-3, Marco5, and Marco7 in the control group (Fig. 3B). Interestingly, Marco5 showed a higher proportion in the G2M phase, indicating its greater proliferative capacity (Supplementary Fig. S3A).

Macrophages are commonly classified into two canonical subtypes, proinflammatory M1 and anti-inflammatory M2 [18]. However, based on common markers such as *Cd68*, *Tspo*, and *Ly6a* (M1), we could not clearly distinguish M1 macrophages (Fig. 3D). Nevertheless, M2 markers, including Cd163, Msr1, and Fcgr1, were highly expressed in Marco4 and Marco6, suggesting their association with tumor progression (Fig. 3D). To gain a better understanding of the roles of Marco4 and Marco6, we further examined the expression levels of evasion-related genes across different subpopulations. We found that almost all evasion-related genes were well expressed in Marco4 and Marco6 (Fig. 3E). Additionally, Marco6 exhibited high expression of *Cd274* (PD-L1), a ligand for PD-1 involved in immune checkpoint regulation in T cells. Moreover, we observed increased signature scores associated with negative regulation of T cell activation pathways (Fig. 3F) and high levels of immunoregulatory genes (Fig. 3G) in Marco4 and Marco6, indicating their higher ability to inhibit T cells. These findings align with the results mentioned earlier (Supplementary Fig. S2D, E).

### Transcriptomic changes of the T lineage in OXA-primed livers

**Fig. 4:**
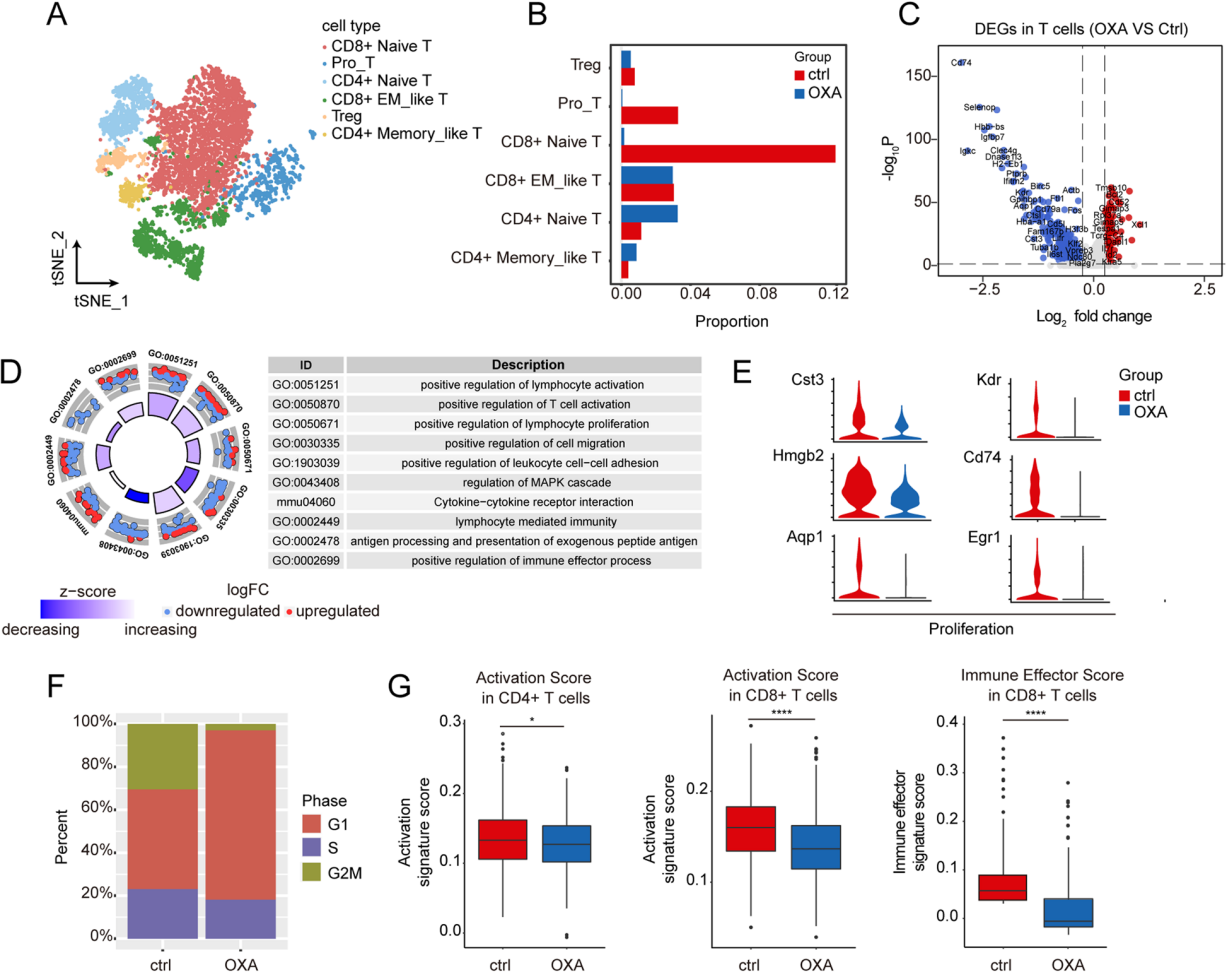
T cell subtypes and their heterogeneity in livers from mice treated with or without OXA. (**A**) t-SNE visualization of 6 subclusters of the T lineage pooled from mice livers. Each cluster is color-coded according to the cell type. Treg, regulatory T cell; Pro-T, proliferating T cells; CD8 + EM_like T, CD8 + effector memory like T. (**B**) Bar plot displaying the changes in the percentage of various subclusters of T cells upon OXA administration. (**C**) Volcano plot showing DEGs of T lineage between unstimulated liver and OXA-treated liver. DEGs were detected by differential expression analysis (two-sided Student’s t-test). Each dot represents a single gene; genes with log2(fold change) (logFC) < -0.25 and logFC > 0.25 were selected and colored in blue and red, respectively. (**D**) Circle plot displaying enrichment analysis of DEGs in T cells, with top 10 enriched terms showed. (**E**) Violin plots showing the expression of genes linked to proliferation T cells upon OXA administration. Representative DEGs enriched in positive regulation of lymphocyte proliferation pathway in (**D**) are displayed. (**F**) Stacked bar chart showing the constitution of different cell cycle phases of T cells with and without OXA treatment based on the average expression of S and G2/M gene sets. (**G**) Box plot showing activation score in CD4 + T cells (left), activation score in CD8 + T cells (middle), and immune effector score in CD8 + T cells (right) from mice livers with and without OXA treatment. AddModuleScore function in Seurat R package was used to calculate the average expression with default settings. *P value < 0.05; ****P value < 0.0001

To gain a better understanding of the transcriptomic changes in T cells that may interact with macrophages, our next objective was to identify expression patterns in T cells following OXA treatment. Through unsupervised clustering analysis, we identified six subtypes based on the expression of cell type-associated genes: CD8 + naïve T cells (*Cd8a* + *Ccr7* +), proliferating T cells (Pro-T, *Mki67* + *Stmn1* +), CD4 + naïve T cells (*Cd4a* + *Ccr7* +), CD8 + effector memory-like T cells (CD8 + EM_like T, *Cd8a* + *Gzmk* +), regulatory T cells (Treg; *Cd4* + *Foxp3* +), and CD4 + memory-like T cells (*Cd4* + *Cd44* + *Icos* + *Gzmk-Gzmb-*) (Fig. 4A, Supplementary Fig. S3B, C). Among these T cell types, CD8 + naïve T cells and Pro-T cells exhibited a decrease in cell percentages upon OXA stimulation (Fig. 4B).

Comparing the transcriptomic features of T cells between the OXA and control groups, we identified 110 upregulated genes and 331 downregulated genes, indicating that OXA induced an immunologically hypo-responsive state in liver T cells (Fig. 4C). Furthermore, pathway enrichment analysis revealed downregulated expression of genes associated with positive regulation of T cell activation, positive regulation of lymphocyte proliferation, and lymphocyte-mediated immunity (Fig. 4D, E). As expected, T cells from mice treated with OXA exhibited a higher proportion in the G1 phase and a lower proportion in the G2M phase, indicating the proliferative status of liver T cells after OXA treatment (Fig. 4F). Additionally, gene scoring analysis revealed a slightly reduced activation score in CD4 + T cells and a significantly reduced activation score and immune effector score in CD8 + T cells, further suggesting the immunologically hypo-responsive features induced by OXA (Fig. 4G).

### The validation of the effects of OXA on T cells in vivo and ex vivo

**Fig. 5:**
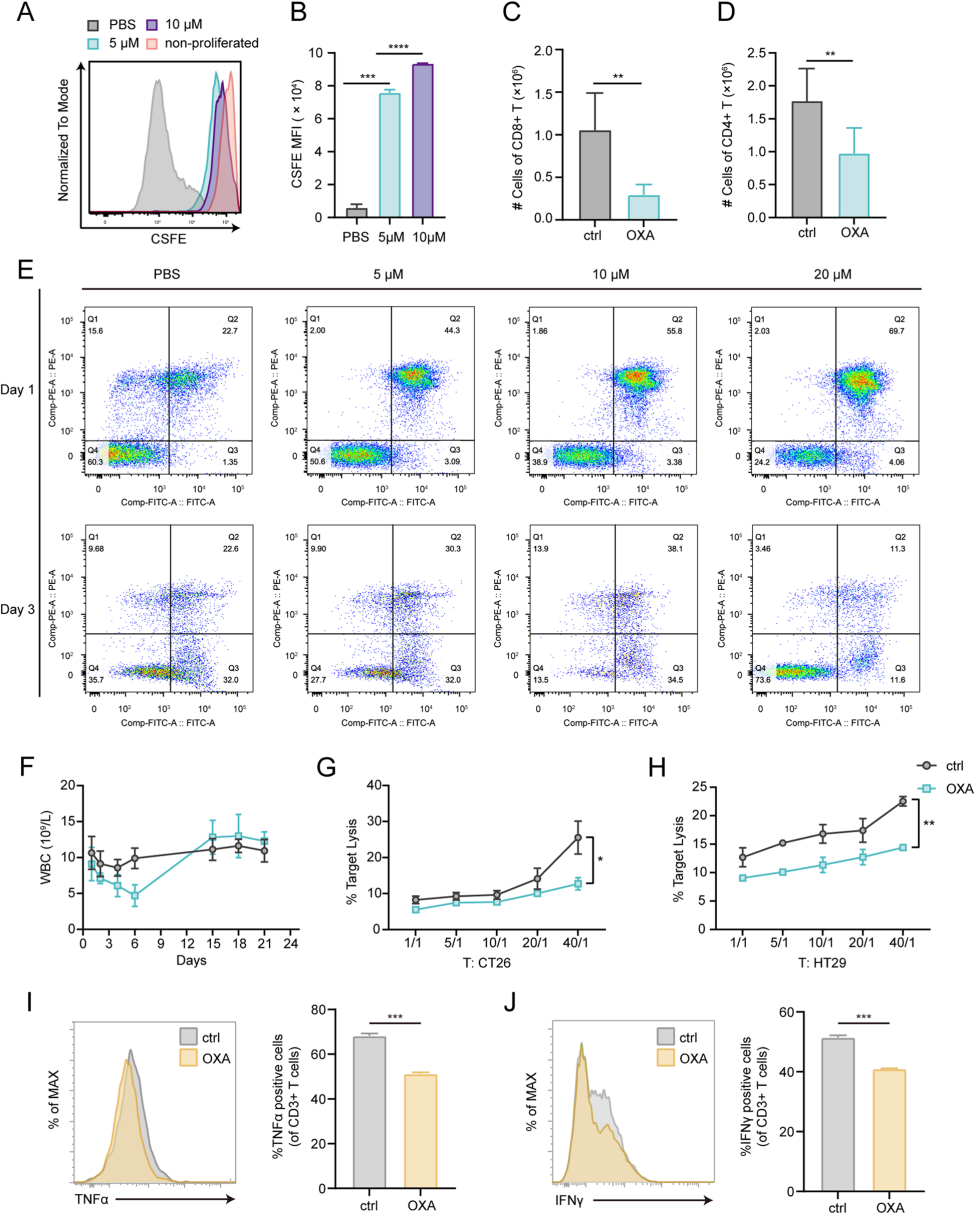
The effects of OXA on T cells in vivo and ex vivo. (**A**) Representative histogram of the division profile of T cell treated with different concentration of OXA for 3 days. (**B**) Quantification of mean fluorescence intensity (MFI) of CFSE in (A) (*n* = 3, mean ± SD). (**C**, **D**) Flow cytometry qualification of the number of CD8 + (**C**) and CD4 + T cells (**D**) in livers 3 days after OXA or PBS treatment. (**E**) Flow cytometry analysis of apoptosis in T cells treated with different concentration of OXA on day 1 and day 3. (**F)** Blood routine test displaying the changes of white blood cell (WBC) absolute number in mice following OXA treatment (*n* = 6 to 8 mice, mean ± SD). (**G**, **H**) Killing assay (10 h) showing the lysis proportion of CT26 cells (**G)** and HT29 cells (**H**) co-cultured with untreated and OXA-treated murine CTLs and human CTLs in different CTL-to-target ratio, respectively. (**I**, **J**) Representative flow-cytometry plots and its qualification, showing the frequency of TNFα + (**I**) and IFNγ + (**J**) T cells co-cultured with macrophages from livers of mice in control and OXA group at 72 h post-activation (*n* = 3). Significant differences were assessed using one-way ANOVA and two-tailed unpaired Student’s t test. *p < 0.05, ** *P* < 0.01, ***P value < 0.001, ****P value < 0.0001

T cells were labeled with CFSE, incubated with increasing concentrations of OXA (0 to 10 µM) for 3 days, and analyzed for proliferation. As depicted in Fig. 5A and B, the presence of OXA resulted in a dose-dependent decrease in cell proliferation activity. Furthermore, the reduction of CD8 + T cells in the liver was observed in mice subjected to OXA administration, and similar results were found for CD4 + T cells (Fig. 5C, D). As anticipated, OXA induced evident apoptosis of T cells in a dose-dependent manner (Fig. 5E).

Given the broad range of effects of chemotherapeutic agents on the organism, we then examined the changes in immune cell numbers and composition in peripheral blood and lungs of mice following OXA administration. We conducted routine blood tests for 3 weeks after OXA administration and observed a decrease in the numbers of white blood cells, lymphocytes, neutrophils, and intermediate cells within the first week (Fig. 5F, Supplementary Fig. S4A, B). Additionally, OXA treatment reduced the number of various immune cells, including T cells, macrophages in the lungs, which exhibited similar results to those observed in the livers (Supplementary Fig. S4C, D). Interestingly, the trends in the proportion of macrophages and monocytes in the lungs after OXA treatment were not observed in the livers (Supplementary Fig. S4E).

To determine whether T cells exhibited lower cytotoxic activity after OXA stimulation, we incubated cancer cells with OXA-primed T cells. After pre-treatment with OXA for 3 days, T cells demonstrated diminished cytotoxicity against murine cell lines CT26 and human cell lines HT29 within 10 h of co-culture (Fig. 5G, H). To investigate whether macrophages derived from OXA-treated mice exerted an inhibitory effect on T cell activation, we isolated liver macrophages and co-cultured with T cells for 72 h (Supplementary Fig. S4F). Consistent with the results of scRNA-seq analyses, the proportion of M2 macrophages in the livers increased following OXA administration (Supplementary Fig. S4G). Subsequently, we employed flow cytometry to characterize the capability of cytokine production of T cells, which reflects their activation and function. Notably, T cells demonstrated a diminished capacity for cytokine secretion upon co-culture with macrophages primed by OXA treatment in vivo (Fig. 5I, J).

### T cells transfusion treatment partially abolishes OXA-induced liver metastases

**Fig. 6:**
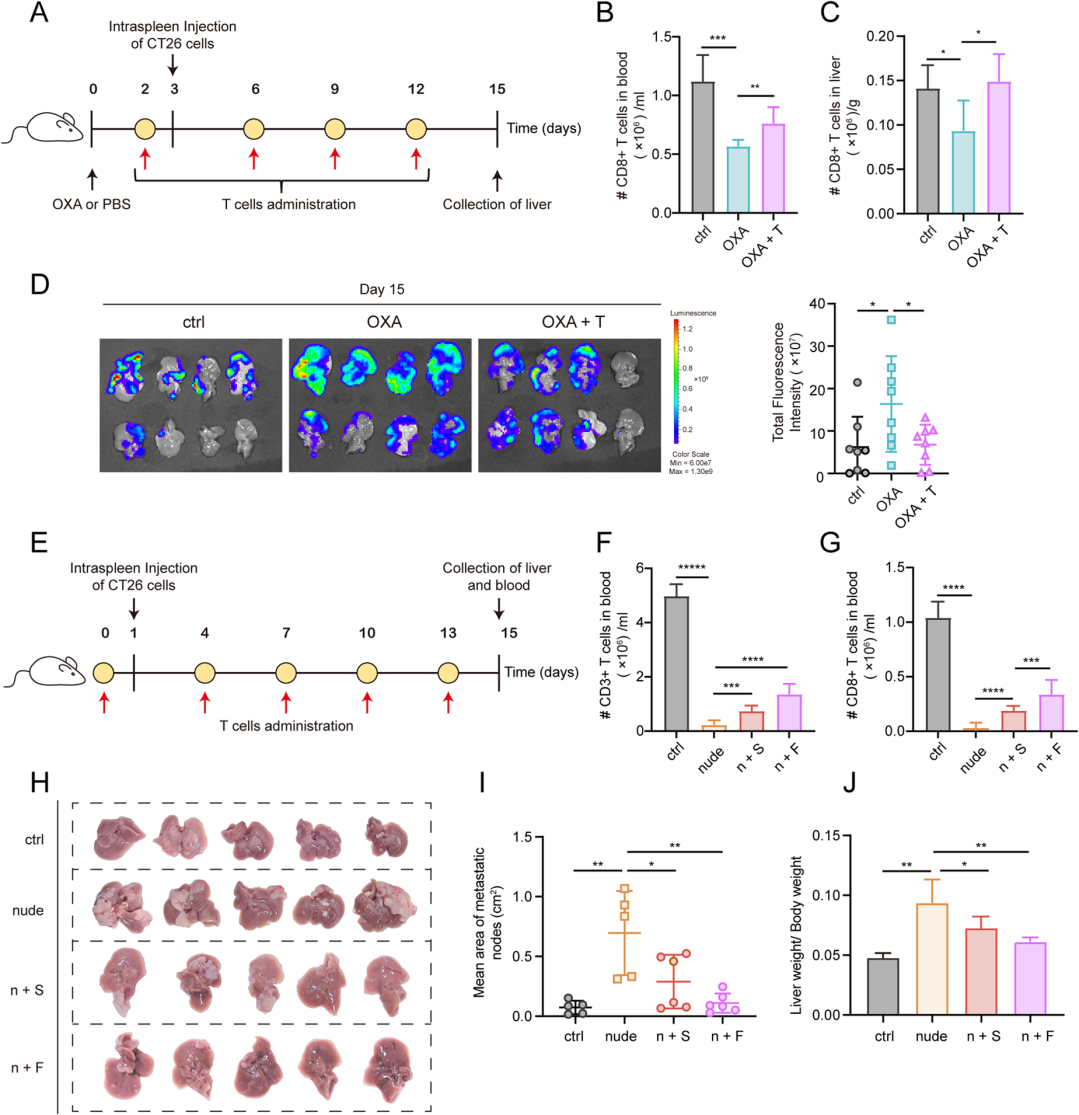
T cells transfusion treatment can effectively abolish the progression in OXA pre-treatment liver metastases model. (**A**) Schematic diagram of OXA administration and T cells infusion in CT26luc liver metastasis model using BALB/c mice. Mice were divided into three groups randomly and administered with PBS (ctrl), OXA (OXA and OXA + T) at first. Subsequently, mice from OXA + T group was treated with forth T cells infusion at the time indicated, with other two group treated with PBS at the same time. (**B**, **C**) Flow cytometry qualification of the number of CD8 + T cells in blood (**B**) and livers (**C**) on day 3 in different groups (mean ± SD, *n* = 8 to 10 mice). (**D**) Luminescence intensities of CT26luc liver metastasis from mice treated with PBS, OXA, and OXA combined T infusion on day 15. Left: Representative liver images. Right: Dot plot showing the metastatic burden in the liver (mean ± SD, *n* = 8 to 10 mice). (**E**) Experimental design for assessing the effects of T cells deficiency and T cells infusion on metastatic burden. Mice were divided into four groups randomly. BALB/c mice (ctrl) and BALB/c-nude mice (nude, n + S, n + F) were injected with CT26luc cells on day 1. For T cells infusion treatment, BALB/c-nude mice were injected intravenously with T cells once (n + S) on day 0 or fifth (n + F) at the time displayed. (**F**, **G**) Flow cytometry qualification of the number of CD3 + ( **F**) and CD8 + T cells (**G**) in blood on day 15 in different groups (mean ± SD, *n* = 5 to 6 mice). (**H**) Representative liver images of metastatic burden in different groups. (**I**) Quantitative analysis of mean area of metastatic nodes in (**H**). (**J**) Metastasis indexes showing metastatic burden in different groups. Significant differences were assessed using one-way ANOVA test. *P value < 0.05, **P value < 0.01, ***P value < 0.001, ****P value < 0.0001

Considering the decrease in absolute number of T cells and the trend towards low responsiveness in transcriptome features following OXA treatment, we hypothesized that supplementing T cells with robust functionality might contribute to suppressing OXA-induced liver metastasis. To test this hypothesis, we infused T cells back into mice one day prior to intrasplenic inoculation of CT26 cells, aiming to modify the OXA-primed liver microenvironment. The mice received T cell infusions three times every other two day after CT26 cell implantation, and were subsequently sacrificed for hepatic metastasis assessment (Fig. 6A). As anticipated, the levels of CD8 + T cells in the liver and peripheral blood partially recovered following T cell infusion treatment (Fig. 6B, C). Furthermore, immunofluorescence analysis of the liver revealed an elevation in the count of CD45 + immune cells and T cells subsequent to T cell infusion therapy, while there were no apparent alterations observed in the macrophage populations (Supplementary Fig. S5A-C). Notably, acceptance of T cell infusion after OXA treatment resulted in a significant decrease in the number of surface liver metastases upon intrasplenic inoculation of CT26 cells (Fig. 6D). To further validate these results and explore the role of T cells in liver metastasis in our OXA-primed model, we utilized immunodeficient mice (BALB/c-nude mice). These mice, lacking T cells, exhibited an increase in hepatic metastatic outgrowth. However, infusion of T cells prior to CT26 cell inoculation led to a reduction in liver metastasis, with the inhibitory effects of T cell treatment becoming more pronounced with an increased frequency of infusion (Fig. 6H-J). Consistently, we also observed a detectable increase in CD3 + T cells and CD8 + T cells in the blood of mice that received T cell infusion (Fig. 6F, G). In summary, our findings suggest that T cell infusion treatment modifies the liver microenvironment induced by OXA prior to the occurrence of liver metastasis and ultimately contributes to the suppression of liver metastasis.

### Relationship between chemotherapy-induced alterations in immune profiles and clinical implications

Consistent with the observed decrease in T cells in mice treated with OXA, previous studies have described lymphopenia as a consequence of antineoplastic chemotherapies [19, 20]. Treatment-related lymphopenia has been identified as a poor prognostic factor in various cancers, including lung [21], colorectal [19], and pancreatic [22] cancers, among others. To investigate the effects of lymphopenia on the prognosis of cancer patients, we conducted a meta-analysis. We collected a total of 5,813 records from PubMed, Embase, Cochrane Library, and Web of Science (Supplementary Figure S6). After excluding 1,749 duplicate records and screening titles and abstracts, we identified 117 relevant papers for further analysis. Subsequently, we excluded 16 review studies, 63 studies lacking posttreatment lymphopenia information, and 39 studies where chemotherapy was not included as an intervention, ultimately including 12 studies in our analysis [19, 21–31]. The clinical information of each study is listed in Supplementary Table 1, and the NOS quality assessment of each study is provided in Supplementary Table 3.

**Fig. 7:**
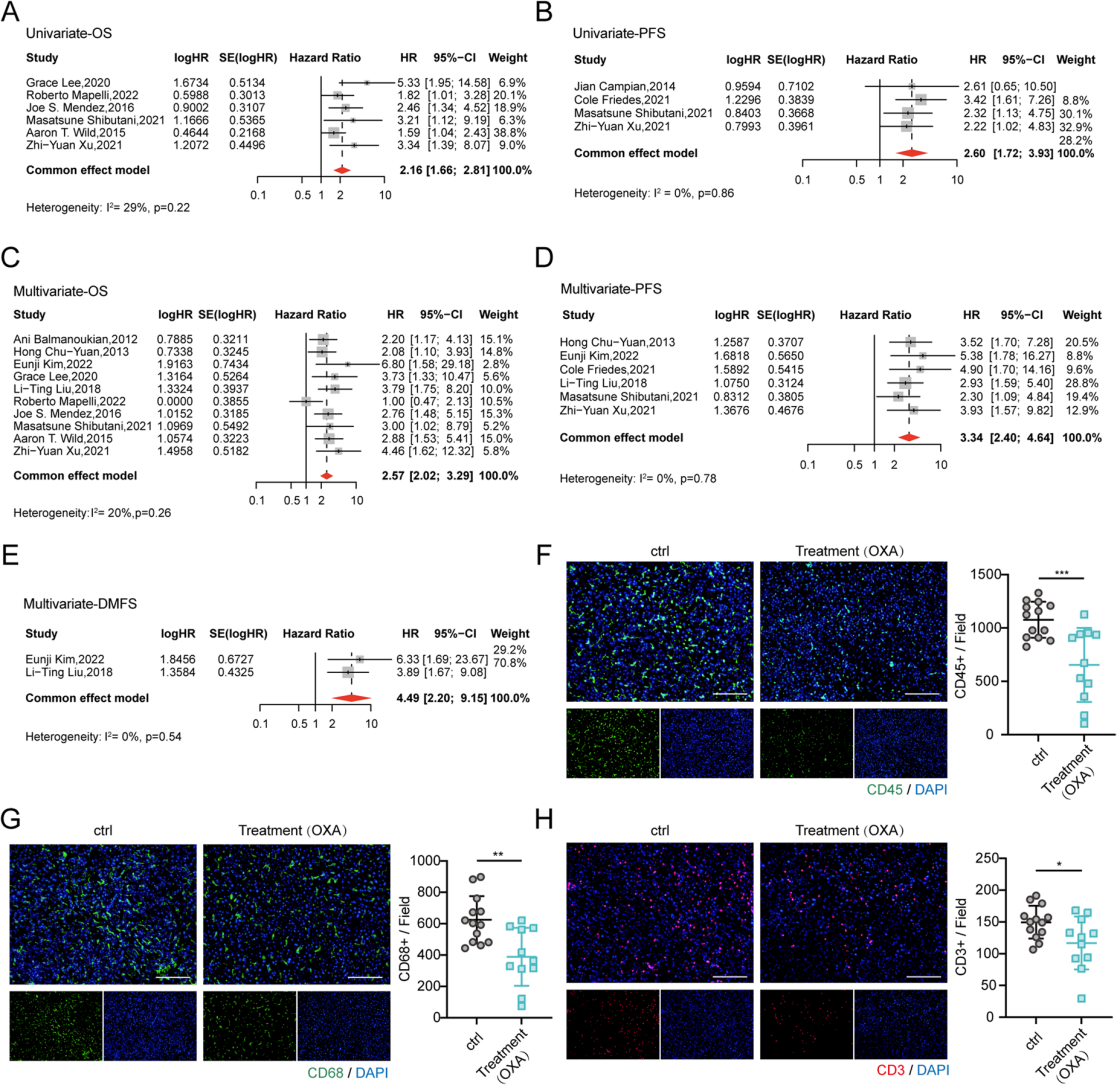
The connection between chemotherapy-related lymphopenia and cancer prognosis and alterations in immune profiles caused by chemotherapy. (**A**, **B**) Forest plots depicting univariate meta-analysis on the association between chemotherapy-related lymphopenia and the OS (**A**), and PFS (**B**) of cancer patients. The large diamond at the bottle of the plot represents the pooled HR of all studies. The width of the diamond represents with 95% CI. (**C**-**E**) Forest plots displaying multivariate meta-analysis on the association between chemotherapy related lymphopenia and the OS (**C**), PFS (**D**), and DMFS (**E**) of cancer patients. The large diamond at the bottle of the plot represents the pooled HR of all studies. The width of the diamond represents with 95% CI. (**F**–**H**) Representative immunofluorescence analyses of CD45 (left, **F**), CD68 (left, **G**) and CD3 (left, **H**) in livers from patients treated with OXA-based chemotherapy or not and quantification analyses of positive cells displayed in the right (at least three random fields were selected, mean ± SD, Scale bars, 100 µm). Significant differences were assessed using one-way ANOVA and two-tailed unpaired Student’s t test. *p < 0.05, **P < 0.01, ***P < 0.001

Both univariate meta-analysis (HR = 2.16, 95%CI = 1.66–2.81, I^2^ = 29%; 6 studies with 592 samples; Fig. 7A and Supplementary Table 2) and multivariate meta-analysis (HR = 2.57, 95%CI = 2.02–3.29, I^2^ = 20%; 10 studies with 1,473 samples; Fig. 7C and Supplementary Table 2) indicated that lymphopenia was associated with inferior overall survival (OS). Univariate meta-analysis (HR = 2.60, 95%CI = 1.72–3.93, I^2^ = 0%; 4 studies with 356 samples; Fig. 7B and Supplementary Table 2) and multivariate meta-analysis (HR = 3.34, 95%CI = 2.40–4.64, I^2^ = 0%; 6 studies with 1,160 samples; Fig. 7D and Supplementary Table 2) also demonstrated that lymphopenia affected the progression-free survival (PFS) of patients. Furthermore, multivariate meta-analysis revealed that compared to OS and PFS, lymphopenia implied a higher risk in distant metastasis-free survival (DMFS) (HR = 4.49, 95%CI = 2.20–9.15, I^2^ = 0%; 2 studies with 561 samples; Fig. 7E and Supplementary Table 2), indicating the close relationship between lymphopenia and metastasis.

In terms of prognostic analysis, sensitivity analysis indicated that excluding one study [26] or another [30] significantly reduced the heterogeneity in univariate meta-analysis of OS, while the HR value remained relatively unchanged (Supplementary Fig. 7A). Similarly, sensitivity analysis in multivariate meta-analysis of OS found that removing one study [28], which potentially contributed to heterogeneity, significantly decreased heterogeneity and increased the HR value (Supplementary Fig. 7C). Regarding PFS in both univariate and multivariate meta-analysis, as well as DMFS in multivariate meta-analysis, sensitivity analysis did not identify the source of heterogeneity (Supplementary Fig. 7B, D-E). The Begg's test indicated no publication bias in this analysis (Supplementary Table 2).

In order to determine whether a similar phenomenon occurs in CRC patients, we enrolled a cohort of 24 individuals diagnosed with liver metastases. Comprehensive preoperative chemotherapy details are provided in Table 1. We analyzed differences in total immune cells, macrophages and T cells, between groups of patients with or without OXA-based chemotherapy before surgery. Notably, a pronounced reduction in the absolute count of CD45^+^ cells, macrophages, and CD3^+^ T cells was evident within the group subjected to OXA-based chemotherapy (Fig. 7F-H).

**Table 1:** Clinical details and neoadjuvant regimens of the included patients

Patient ID	Age (y)	Gender	Neoadjuvant regimens
P01	63	Male	—
P02	58	Male	—
P03	61	Male	—
P04	63	Male	—
P05	55	Male	—
P06	71	Female	—
P07	70	Male	—
P08	63	Female	—
P09	61	Female	—
P10	58	Male	—
P11	67	Male	—
P12	72	Male	—
P13	61	Male	—
PX01	42	Male	IROX + Bevacizumab
PX02	74	Male	XELOX
PX03	39	Male	FOLFOX
PX04	62	Female	XELOX
PX05	47	Female	XELOX
PX06	63	Male	IROX
PX07	63	Male	FOLFOX
PX08	68	Male	XELOX
PX09	64	Female	XELOX + Bevacizumab
PX10	75	Male	XELOX
PX11	64	Female	XELOX

*IROX* Oxaliplatin + Irinotecan, *XELOX* Oxaliplatin + Capecitabine, *FOLFOX* Fluorouracil + Calcium + levofolinate + Oxaliplatin

Collectively, clinical data revealed that treatment-associated lymphopenia was associated with a worse prognosis in cancer patients. Particularly, patients with lymphopenia have a higher risk of metastasis formation, which is consistent with the observations in our mouse model of liver metastasis. These findings further confirm the critical role of T cells in the progression of metastasis. Furthermore, the phenomenon of diminished macrophage and T cell numbers, as observed in the murine models, was also evident in humans undergoing OXA-based chemotherapy, which suggested that we should pay attention to paradoxical promotion of metastasis of chemotherapy.

## Discussion

Most studies in pre-metastasis niche focus on the microenvironment of metastatic organ shaped by primary tumor through secretion of exosomes containing either microRNA or protein [32–34]. Chemical drugs, the main side effect inducer of cancer therapy, have not been investigated in this aspect, although such study will be very important for clinical practice. Oxaliplatin (OXA) has been reported to induce sinusoidal injury in the liver in 19% to 52% of patients [35–38]. Whether this injury contributes to the formation of pre-metastasis niche worth investigation since OXA is a crucial chemotherapeutic agent used for treating colorectal liver metastases (CRLM).

To investigate the effect on liver metastasis by OXA we developed a mouse model with a pre-treatment of OXA before tumor inoculation. This approach allowed us to examine the effects of chemotherapy on the metastatic microenvironment in the absence of primary cancer. Our results demonstrated a significant enhancement of liver metastasis of colon cancer if the mice were pre-treated with OXA. The further experiments revealed a change of immune landscape modified by OXA including a reduction in the total number of T cells in the liver and rendered T cell hypo-responsive to cancer cells. Importantly, the increased liver metastasis induced by OXA was markedly suppressed by the infusion of T cells one day before cancer cell administration, suggesting that the OXA-primed changes in the liver microenvironment could be repaired through T cell supplementation. These findings highlight the need to balance the possibility of metastasis formation and the anti-cancer benefits of OXA treatment.

Recent evidence suggests that chemotherapy can paradoxically induce a pro-metastatic tumor microenvironment, potentially obscuring the long-term benefits of anti-cancer therapies [39]. For example, previous study has shown that ovarian tumor cell debris generated by platinum- and taxane-based chemotherapy triggers a macrophage-derived surge of pro-inflammatory cytokines, contributing to the pro-metastatic tumor microenvironment [40]. Similarly, gemcitabine-educated mesenchymal stem cells (MSCs) have been found to support the cancer stem cell compartment and enhance tumor growth [41]. These studies have primarily focused on the pro-metastatic effects mediated by chemotherapy in the presence of cancer cells. In contrast, our study extends the concept by demonstrating that chemotherapy-induced re-programming of the metastatic microenvironment can occur even in the absence of a primary CRC tumor. This suggests that the host response elicited by chemotherapy may interfere with the benefits of the therapy.

Initially, we explored the connection between OXA-induced liver injury and enhanced liver metastasis. However, we did not detect significant pathological changes nor in serum levels of ALT and AST, indicating that a single injection of OXA in our model was insufficient to cause detectable liver damage. To further understand the underlying mechanism, we performed scRNA-seq to examine the immune microenvironment in the liver elicited by OXA. Consistent with the liver injury results, we did not observe significant changes in genes related to vascular injury [42]. However, we did find a decrease in the number of immune cells, particularly macrophages and T cells, in the OXA-treated group. Studies have shown that chemotherapy can alter the polarization of intratumoral macrophages, promoting a pro-tumor M2-like phenotype [43–45]. We identified changes in the polarization status of liver macrophages based on scRNA-seq data, with two subtypes of macrophages exhibiting M2-like phenotype and high ability to inhibit T cell activation. Inspired by this observation, we focused our subsequent investigations on T lymphocytes, which play a key role in defense against pathogens and cancer cells [46]. Transcriptome analysis revealed downregulation of pathways associated with proliferation, activation, and immune effector processes in T cells, particularly CD8^+^ T cells, indicating OXA-induced immune hypo-responsiveness in liver T cells. There have been reported several factors, such as high expression of SOCS1 in dendritic cells [47, 48], production of anti-inflammatory cytokines like interleukin-10 by macrophages [49], and intact mitochondrial translation function [50], contributing to T cell hypo-responsiveness and reduced killing capacity. Further investigations are required to elucidate the potential mechanisms underlying OXA-induced T cell hypo-reactivity and the detailed interaction between macrophages and T cells. Moreover, co-culture assays revealed that the activation of T cells was inhibited when co-cultured with macrophages from mice receiving OXA treatment. In addition, in vivo and ex vivo validations suggest that the changes in T cells induced by OXA were not solely dependent on the interaction with macrophages.

Recent studies have demonstrated that chemotherapy-related lymphopenia is closely linked to poor prognosis in various cancers, including diffuse large B-cell lymphoma [20], resected pancreatic adenocarcinoma [22], colorectal cancer [24], and squamous cell carcinoma of the anal canal [26]. Our meta-analysis of clinical data has confirmed the impact of chemotherapy-related lymphopenia on cancer patient prognosis. Strikingly, we revealed a negative correlation between lymphopenia and distant metastasis-free survival (DMFS), indicating a higher risk of metastasis formation in patients with lymphopenia. Based on these findings, we hypothesized that the supplementary of immune cells with functionally intact T cells could improve the pro-metastatic microenvironment by OXA. Indeed, infusion of T cells increased the number of CD3^+^ cells in the liver of OXA-treated mice and partially abolished the OXA-induced liver metastasis. The crucial role of T cells in cancer progression was further confirmed by using immune-deficient mice lacking T cells, which exhibited increased hepatic metastatic tumor growth. Notably, a single infusion of T cells before tumor inoculation was sufficient to suppress OXA-elicited metastasis, indicating that infused T cells could modify the OXA-educated microenvironment in the liver, beyond their direct killing effect on tumors. Furthermore, it is noteworthy that a decrease in the absolute count of both macrophages and T cells was also evident among CRC patients undergoing OXA-based chemotherapy. This observation implies a possible facilitation of metastasis formation. Considering the intricate interaction network among different cell types and cytokines, further exploration is necessary to determine whether T cell infusion affects the status of other immune cells. Additionally, the involvement of other immune cells in the construction of the pro-metastatic liver microenvironment is worth further exploration.

## Conclusions

In summary, our study revealed that the application of OXA accompanies a formation of immunosupressive microenvironment in the liver which might contribute to liver metastases. Immunosuppressive macrophages and unresponsive T cells in the liver increased its susceptibility to colonization by CRC cells. Fortunately, infusion of functional T cells significantly reversed the side effects and decreased OXA-induced liver metastases. These findings underscore the need to judge the impact of chemical agents on the metastatic microenvironment, especially in patients who exhibit poor response or resistance to the treatment containing the agent, as it may compromise the therapeutic benefits.

## Supplementary Information

**Additional file 1.**

## Data Availability

The datasets used and/or analyzed during the current study are available from the corresponding author on reasonable request.

## Abbreviations

CRC: Colorectal cancer
OXA: Oxaliplatin
FOLFOX: 5-FU/leucovorin and oxaliplatin
FOLFIRI: 5-FU/leucovorin and irinotecan
CRLM: Colorectal liver metastases
SOS: Sinusoidal obstruction syndrome
EVs: Extracellular vehicles
ALT: Alanine Aminotransferase
AST: Aspartate Transaminase
scRNA seq: Single-cell RNA Sequencing
t-SNE: T-distributed stochastic neighbor embedding
DEGs: Differentially expressed genes
FDR: False discovery rate
HR: Hazard Ratio
OS: Overall survival
PFS: Progression-free survival
DMFS: Metastasis-free survival
