# Correction: Impact of chemotherapeutic agents on liver microenvironment: oxaliplatin create a pro-metastatic landscape

**DOI:** 10.1186/s13046-025-03604-3

**Published:** 2025-12-27

**Authors:** Yuanyuan Ma, Chang Guo, Xijun Wang, Xundong Wei, Jie Ma

**Affiliations:** 1 Center of Biotherapy, Beijing Hospital, National Center of Gerontology, Institute of Geriatric Medicine, Graduate School of Peking Union Medical College, Chinese Academy of Medical Sciences, Beijing, 100730 People’s Republic of China; 2 Savaid Medical School, University of Chinese Academy of Sciences, Beijing, 100049 People’s Republic of China

**Correction: J Exp Clin Cancer Res 42**,** 237 (2023)**

**https://doi.org/10.1186/s13046-023-02804-z**

Following the publication of the original article [1], the authors identified an error in the Ethics project approval number. The correct approval number is highlighted below in **bold**.

**Current Ethics approval and consent to participate**:

Animal studies were performed in accordance with the guidelines of the Laboratory Animal Ethics Committee of Cancer Hospital Chinese Academy of Medical Sciences. The use of pathological specimens and the review of all patient clinical records were approved by the Ethics Committee of National Cancer Center/ Cancer Hospital, Chinese Academy of Medical Sciences and Peking Union Medical College (project approval number 21/329–3000).

**Corrected Ethics approval and consent to participate**:

Animal studies were performed in accordance with the guidelines of the Laboratory Animal Ethics Committee of Cancer Hospital Chinese Academy of Medical Sciences. The use of pathological specimens and the review of all patient clinical records were approved by the Ethics Committee of National Cancer Center/ Cancer Hospital, Chinese Academy of Medical Sciences and Peking Union Medical College (project approval number **22/130–3331**).

The correction does not compromise the validity of the conclusions and the overall content of the article. The original article [1] has been updated.
