# Supplementary figures and images for: Impact of chemotherapeutic agents on liver microenvironment: oxaliplatin create a pro-metastatic landscape
Source: J Exp Clin Cancer Res. 2023 Sep 11;42:237. doi: 10.1186/s13046-023-02804-z (PMC10494354; doi:10.1186/s13046-023-02804-z)

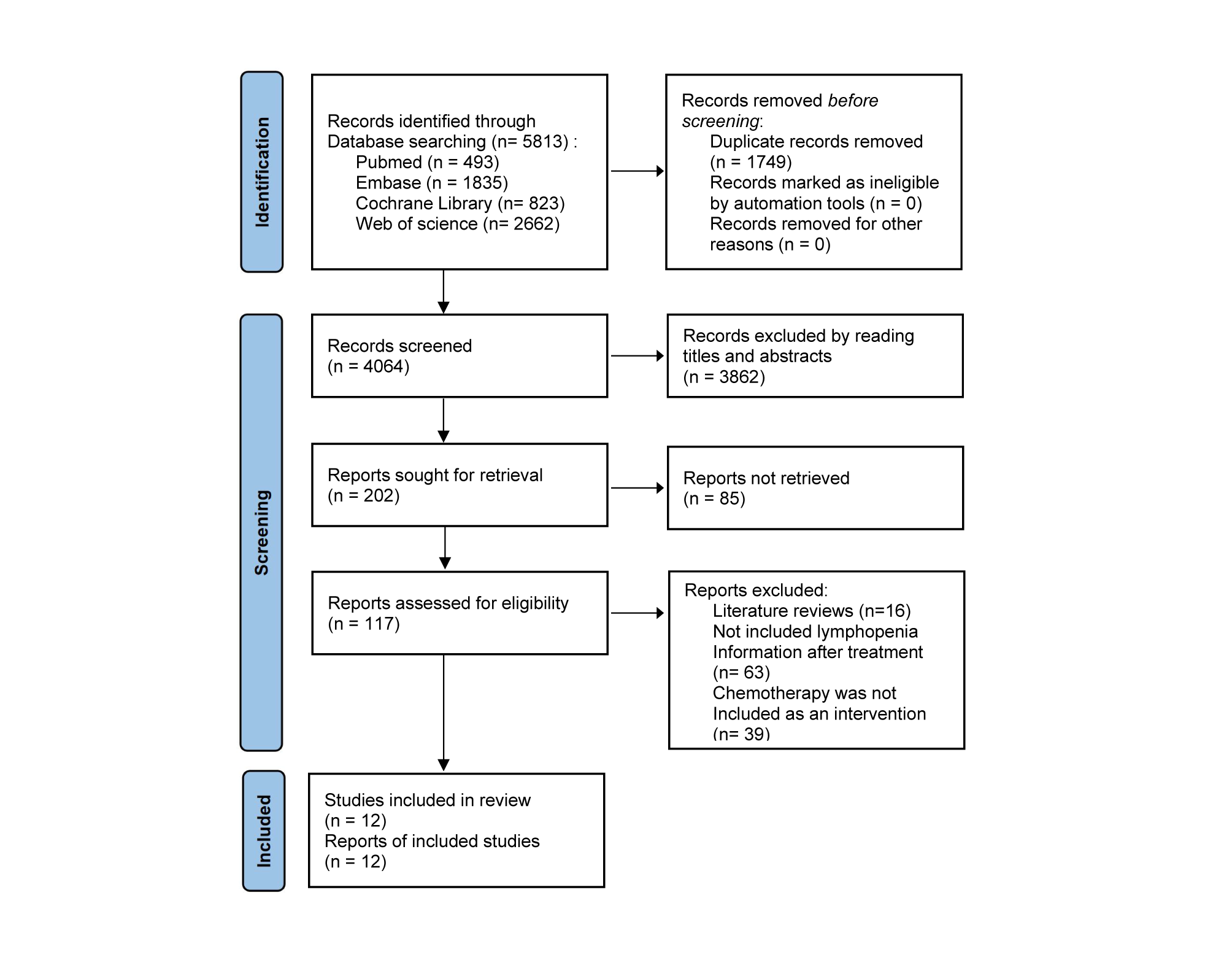

Supplement: Supplementary file 1 — Additional file 1. [file 13046_2023_2804_MOESM1_ESM.zip › S Fig6.tif]

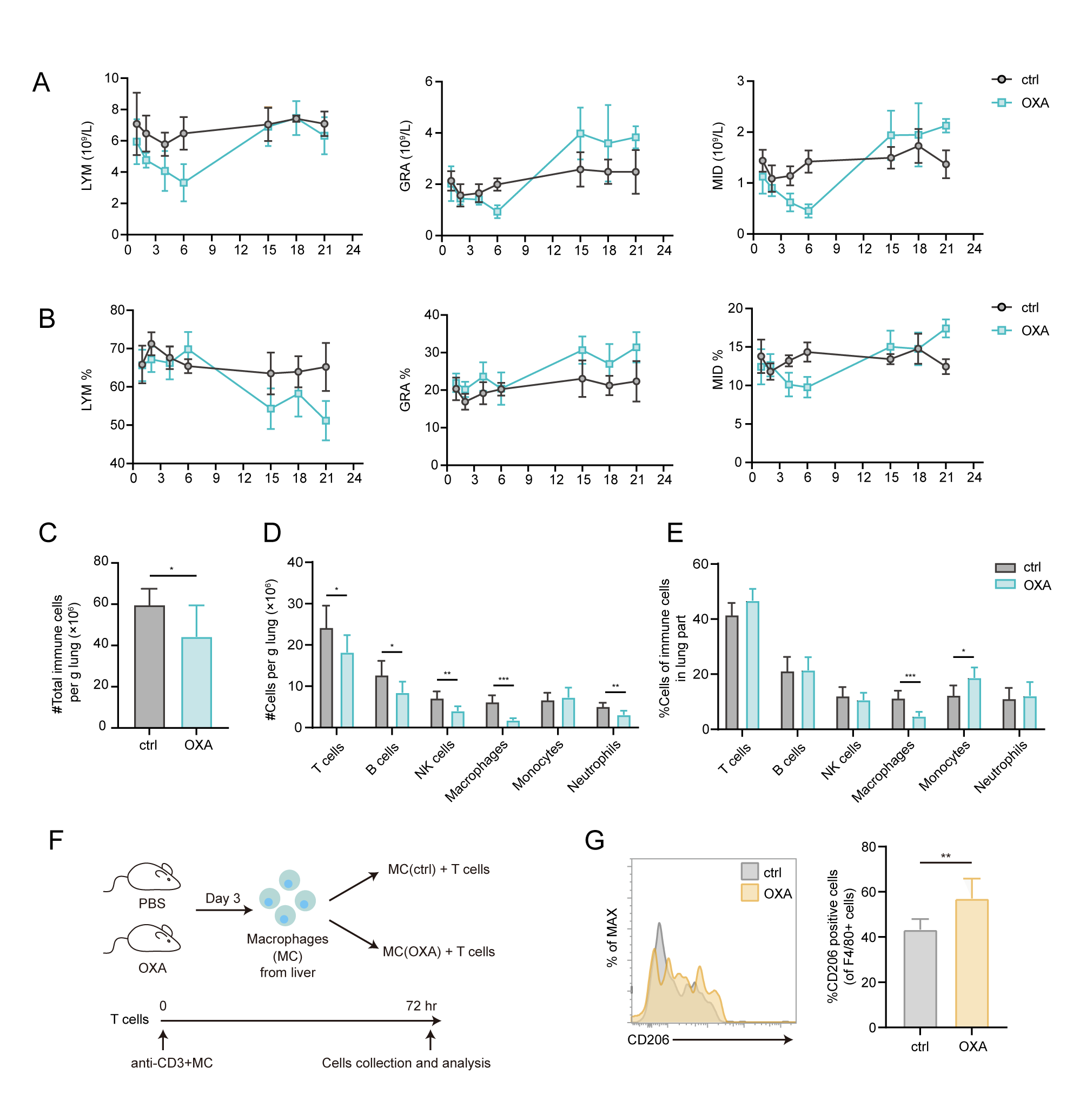

Supplement: Supplementary file 1 — Additional file 1. [file 13046_2023_2804_MOESM1_ESM.zip › S Fig4.tif]

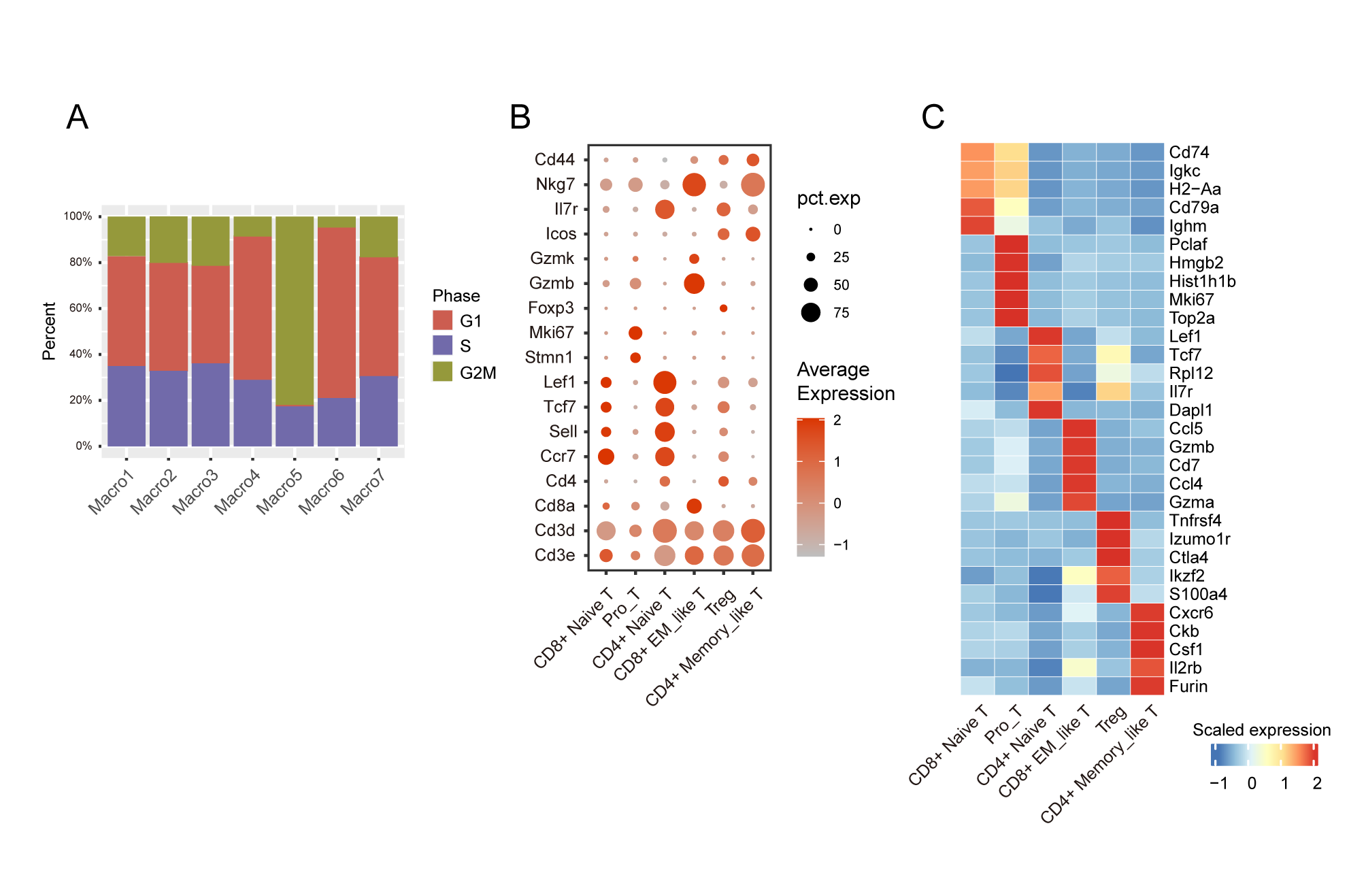

Supplement: Supplementary file 1 — Additional file 1. [file 13046_2023_2804_MOESM1_ESM.zip › S Fig3.tif]

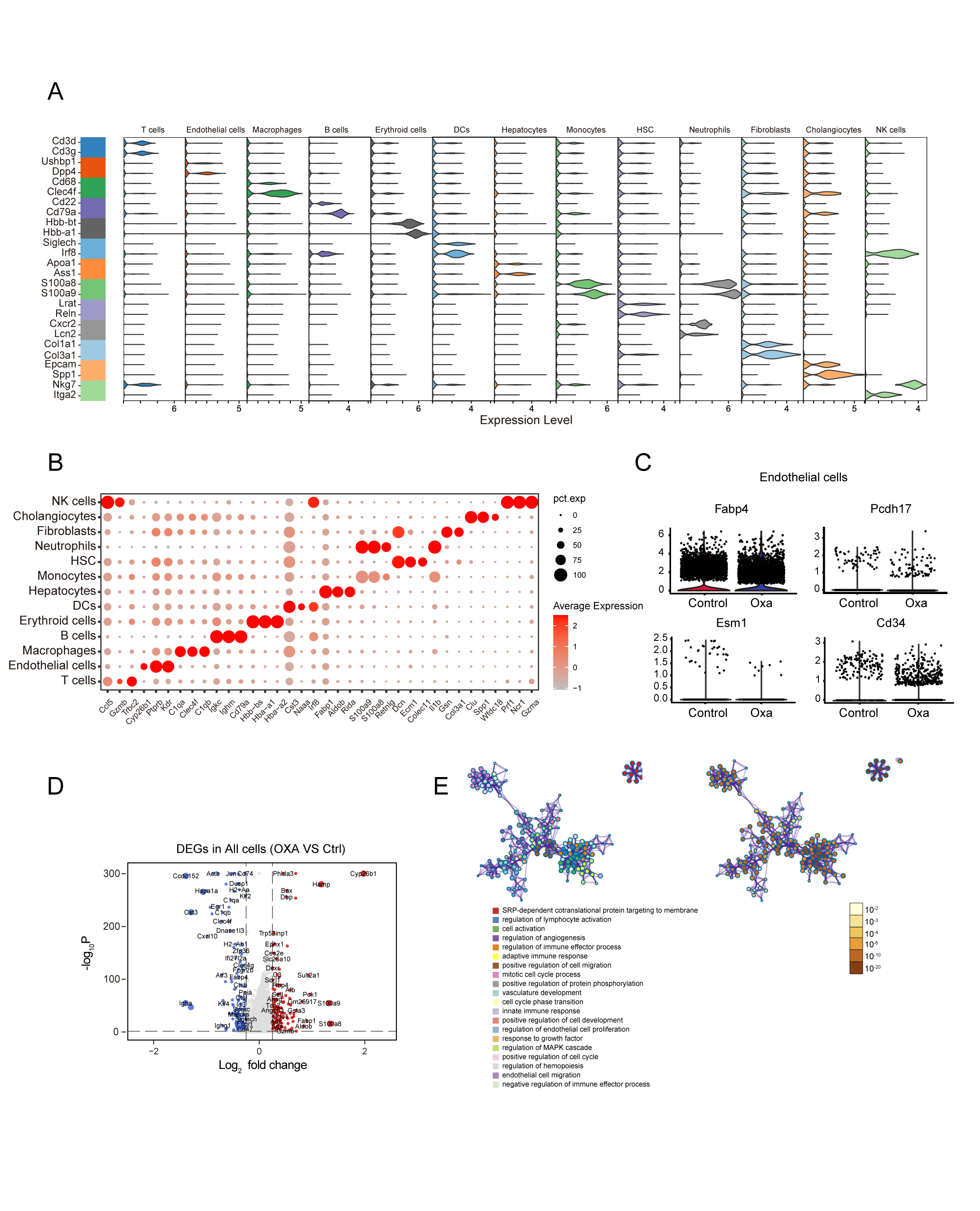

Supplement: Supplementary file 1 — Additional file 1. [file 13046_2023_2804_MOESM1_ESM.zip › S Fig2.tif]

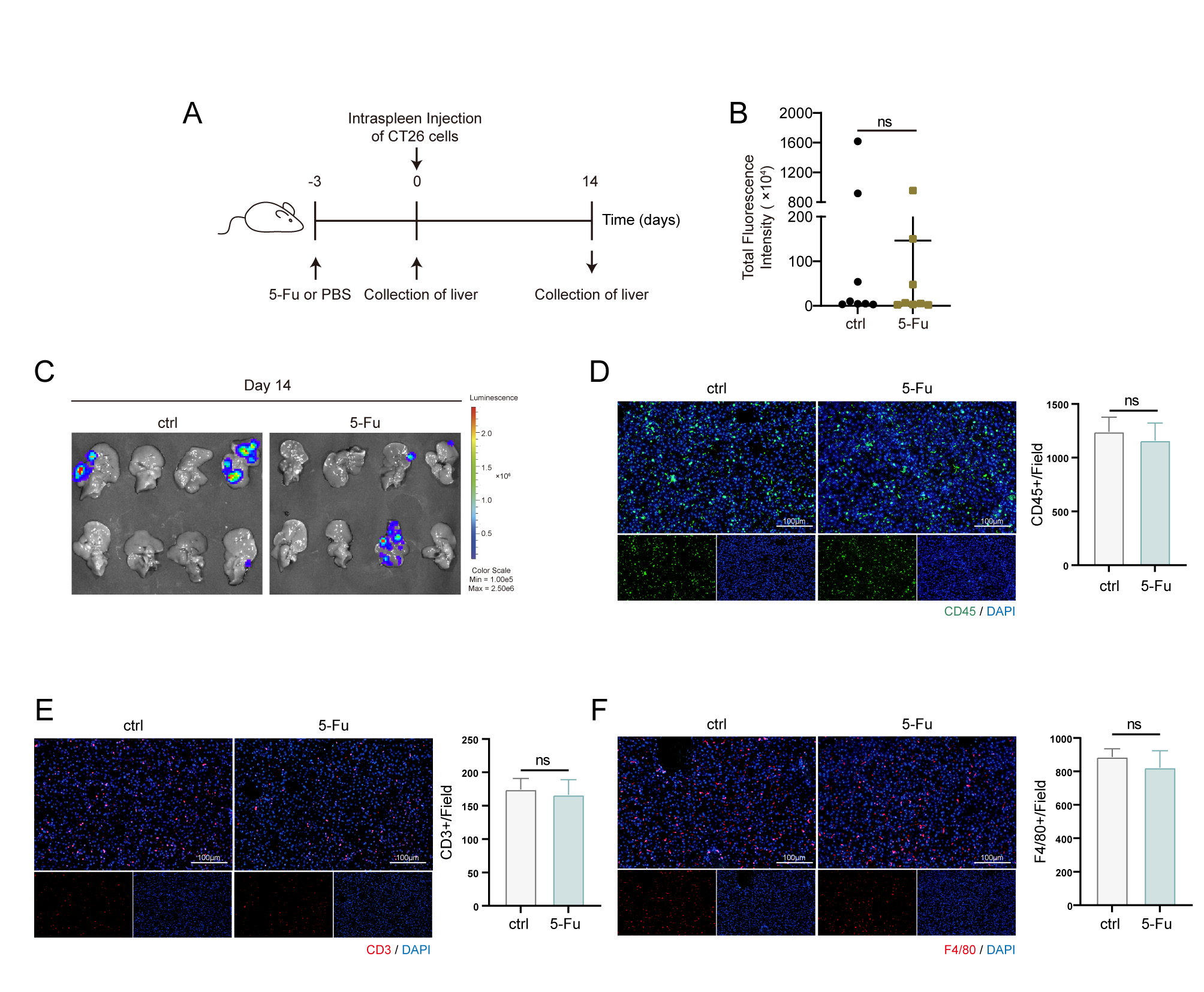

Supplement: Supplementary file 1 — Additional file 1. [file 13046_2023_2804_MOESM1_ESM.zip › S Fig1.tif]

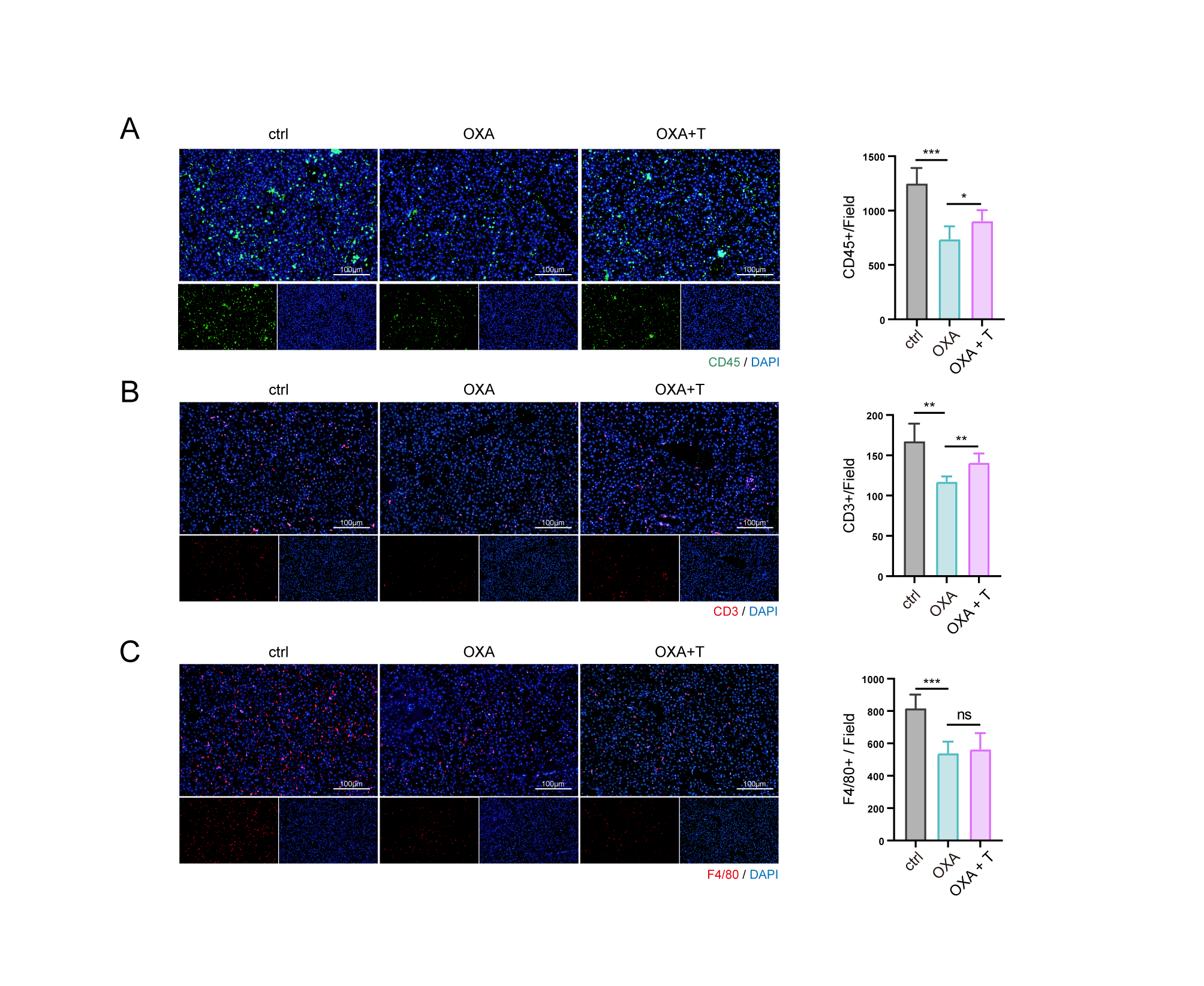

Supplement: Supplementary file 1 — Additional file 1. [file 13046_2023_2804_MOESM1_ESM.zip › S Fig5.tif]

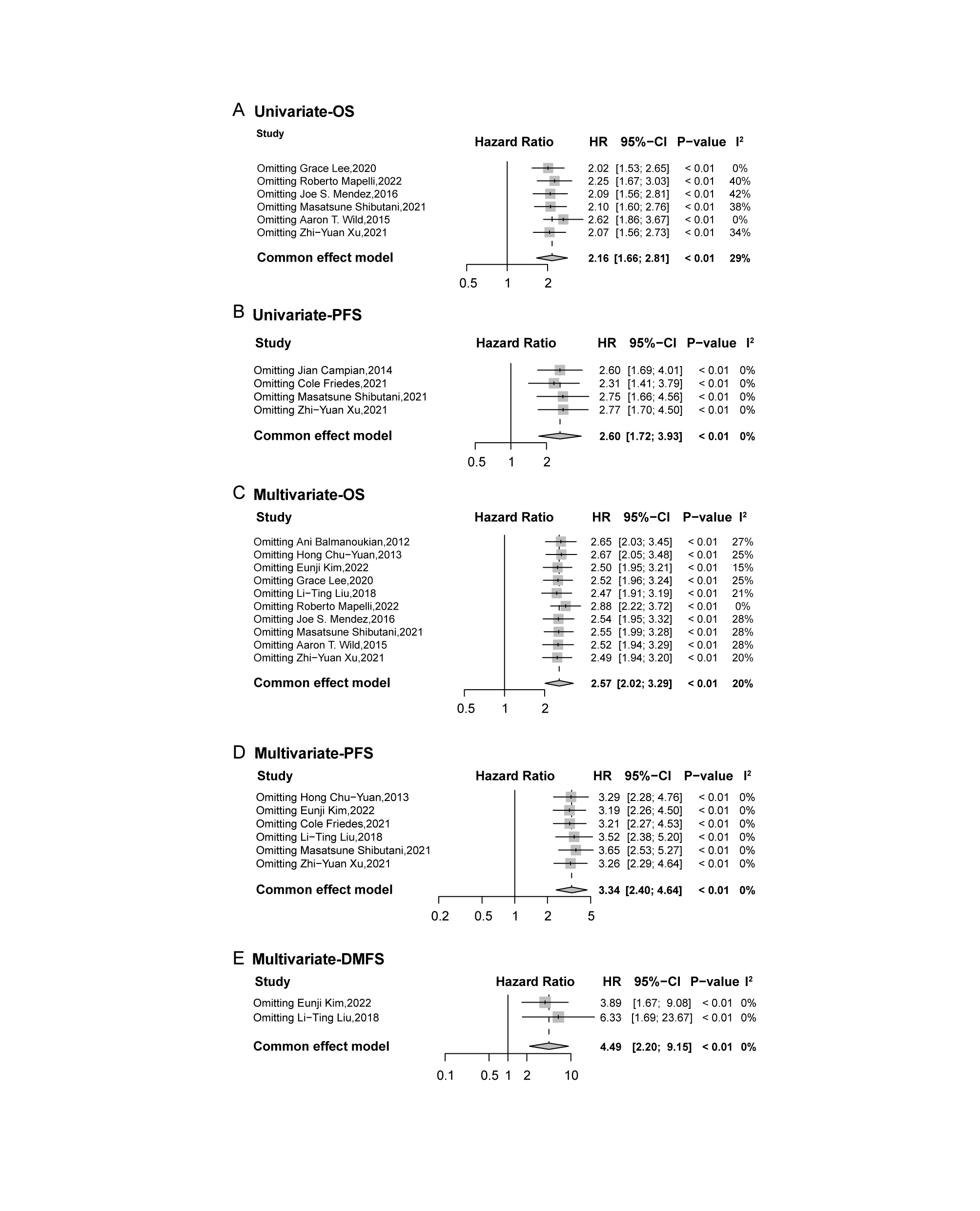

Supplement: Supplementary file 1 — Additional file 1. [file 13046_2023_2804_MOESM1_ESM.zip › S Fig7.tif]
